# Arbuscular mycorrhizal fungal community composition affected by original elevation rather than translocation along an altitudinal gradient on the Qinghai-Tibet Plateau

**DOI:** 10.1038/srep36606

**Published:** 2016-11-09

**Authors:** Wei Yang, Yong Zheng, Cheng Gao, Ji-Chuang Duan, Shi-Ping Wang, Liang-Dong Guo

**Affiliations:** 1 State Key Laboratory of Mycology, Institute of Microbiology, Chinese Academy of Sciences, Beijing 100101, China; 2 College of Resources and Environment, Northeast Agricultural University, Harbin 150030, China; 3 Binhai Research Institute in Tianjin, Tianjin 300457, China; 4 Laboratory of Alpine Ecology and Biodiversity, Institute of Tibetan Plateau Research, Chinese Academy of Sciences, Beijing 100101, China; 5 College of Life Sciences, University of Chinese Academy of Sciences, Beijing 100049, China

## Abstract

Elucidating arbuscular mycorrhizal (AM) fungal responses to elevation changes is critical to improve understanding of microbial function in ecosystems under global asymmetrical climate change scenarios. Here we examined AM fungal community in a two-year reciprocal translocation of vegetation-intact soil blocks along an altitudinal gradient (3,200 m to 3,800 m) in an alpine meadow on the Qinghai-Tibet Plateau. AM fungal spore density was significantly higher at lower elevation than at higher elevation regardless of translocation, except that this parameter was significantly increased by upward translocation from original 3,200 m to 3,400 m and 3,600 m. Seventy-three operational taxonomic units (OTUs) of AM fungi were recovered using 454-pyrosequencing of 18S rDNA sequences at a 97% sequence similarity. Original elevation, downward translocation and upward translocation did not significantly affect AM fungal OTU richness. However, with increasing altitude the OTU richness of Acaulosporaceae and Ambisporaceae increased, but the OTU richness of Gigasporaceae and Glomeraceae decreased generally. The AM fungal community composition was significantly structured by original elevation but not by downward translocation and upward translocation. Our findings highlight that compared with the short-term reciprocal translocation, original elevation is a stronger determinant in shaping AM fungal community in the Qinghai-Tibet alpine meadow.

Elucidating the biodiversity patterns along altitudinal gradients is fundamental to understanding the community assembly and ecosystem functioning^1^,^2^. Previous studies have documented that plant diversity generally either monotonically decreases or has a hump-shaped pattern with increasing altitude^3^,^4^. However, as important components of soil microorganisms, arbuscular mycorrhizal (AM) fungi formed symbiotic associations with most terrestrial plant species, have shown increased, decreased, or hump-shaped diversity patterns with increasing altitude^5^,^6^,^7^,^8^. This divergence in plant and AM fungal altitudinal diversity patterns may be because plant diversity is mainly determined by temperature and dispersal limitation^3^,^4^,^9^; in contrast, AM fungal diversity is influenced not only by plant identity and community^10^,^11^,^12^,^13^, but also by abiotic factors such as soil and climatic variables^8^,^14^,^15^,^16^.

Global climate change is one of the greatest challenges facing our society, and global surface temperature is predicted to increase by 1.8–3.6 °C over the next century^17^. Particularly, high-elevation ecosystems are expected to be sensitive to climate warming as cold temperature constrains biological processes^18^. Temperature manipulation studies have shown that warming affects not only plant productivity, diversity and community composition^19^,^20^, but also AM fungal community structure and function in ecosystems^21^,^22^. For example, experimental warming has neutral or positive effects on AM fungal spore density and species richness in ecosystems^23^,^24^,^25^,^26^,^27^. Warming significantly changes soil AM fungal community composition in an alpine meadow in western China^24^, but not in a grassland in UK^28^ and a semiarid steppe in northern China^25^,^26^. Although artificial heating facilities such as buried electric-resistance heating wires and free air temperature enhancement are widely adopted in field warming experiments, they are regarded as a unsuitable experimental method, especially for long-term research, due to energy-extensive consumption^29^.

Reciprocal translocation experiments that exchange the whole soil-plant along altitudinal gradients provide relatively natural gradient warming and cooling processes, without need for a high energy supply^29^,^30^. Although many environmental variables co-vary with elevation changes (*e.g.* cloudiness, atmospheric density, absolute O_2_ or CO_2_ concentration, ultraviolet radiation, and soil moisture), temperature is considered to be the key driver of variation in ecological processes (*e.g.* plant photosynthesis, productivity and phenology, soil microbial community structure and nitrogen [N] transformation)^20^,^31^,^32^,^33^. Therefore, it is suggested that reciprocal translocation experiment is the most rigorous way of demonstrating impacts of climate change because this experiment allows applying both warming and cooling processes and is believed to reduce chemical and mechanical disturbances to a minimum^29^. Several reciprocal translocation experiments have been conducted on plant survival and productivity^30^, soil properties and respiration^29^,^34^, and soil microbial community^33^,^35^,^36^ in natural grassland ecosystems. However, the AM fungal response to soil-plant reciprocal translocation along an altitudinal gradient remains largely unexplored.

To better understand the effects of original elevation, warming and cooling resulted from elevation changes on AM fungal community, we measured AM fungal spore density under a two-year reciprocal translocation of vegetation-intact soil blocks along an altitudinal gradient in an alpine meadow ecosystem on the Qinghai-Tibet Plateau. The AM fungal community composition in soil was examined using 454 pyrosequencing of 18S rDNA sequecnes. The aim of this study was to investigate how the spore density, diversity and community composition of AM fungi change under reciprocal translocation along an altitudinal gradient in an alpine meadow ecosystem on the Qinghai-Tibet Plateau. The expected results may provide new insights into how AM fungal community would give feedback on global climate change scenarios in the alpine meadow ecosystem.

## Results

### AM fungal spore density

**Figure 1:**
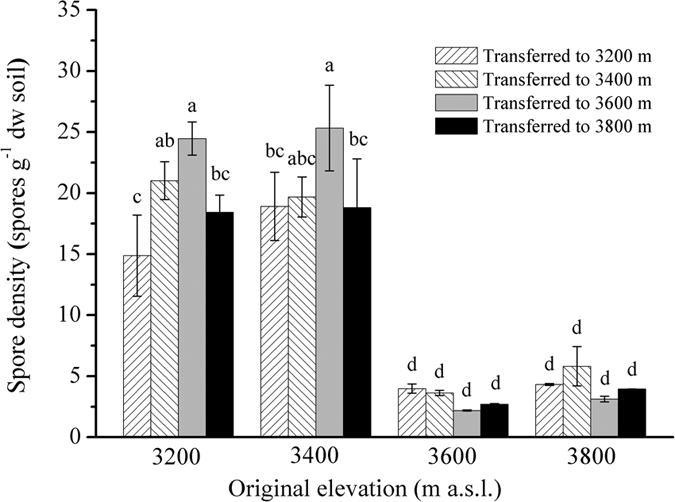
Arbuscular mycorrhizal fungal spore density under reciprocal translocation along an altitudinal gradient. Data are mean ± SE (*n *= 3). Columns without shared letters denote significant difference at *P* < 0.05.

Kruskal–Wallis tests showed that AM fungal spore density was significantly affected by original elevation (*i.e.* translocation to the same elevation) (*X*^2 ^= 36.74, *P *< 0.001), but marginally affected by translocation (*X*^2^ = 12.357, *P* = 0.054). For example, the AM fungal spore density was significantly higher at original 3,200 m and 3,400 m than at original 3,600 m and 3,800 m (Fig. 1). Moreover, the AM fungal spore density was significantly increased by upward translocation from original 3,200 m to 3,400 m and 3,600 m, but not to 3,800 m; whereas this parameter was not significantly affected by downward and upward translocation from original 3,400 m, 3,600 m and 3,800 m to other elevation sites, respectively (Fig. 1). Furthermore, AM fungal spore density was significantly negatively correlated with soil moisture and total organic carbon (TOC) (Supplementary Fig. S1a,b), but positively correlated with plant species richness (Supplementary Fig. S1c).

### Pyrosequencing analysis and identification of AM fungi

A total of 167,899 reads were obtained from 207,569 raw reads after a denoising step, subsequently 18,443 reads were removed through a trimming step. Of the remaining 149,456 reads, 8,862 potential chimeras were removed from the dataset. The remaining 140,594 non-chimeric reads were assigned to 305 operational taxonomic units (OTUs) based on a 97% sequence similarity. Of these 305 OTUs, 73 (42,959 reads) belonged to AM fungi. As the number of AM fungal reads ranged from 144 to 3,249 among the samples, the read numbers were normalized to 144, resulting in a normalized dataset containing 73 AM fungal OTUs (6,912 reads).

**Figure 2:**
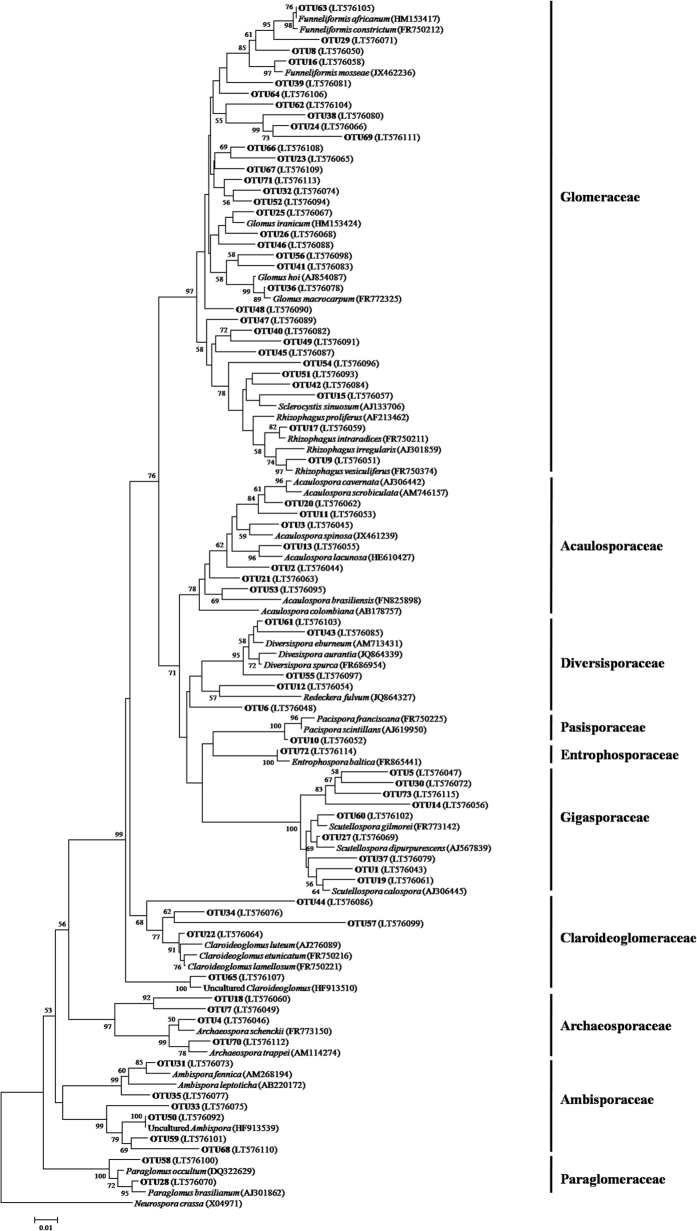
Neighbor-joining tree of arbuscular mycorrhizal (AM) fungi. The 18S rDNA sequences (*ca*. 400 bp) of AM fungal OTUs obtained in this study and the references downloaded from GenBank were shown concomitantly by the corresponding accession numbers (parentheses). Bootstrap values were calculated on the basis of 1,000 data resampling (>50% of the values are shown). *Neurospora crassa* was used as an outgroup. Scale bar represents 1% sequence divergence.

Of these 73 AM fungal OTUs, the first 10 frequent OTUs occurred in ≥24 (50%) soil samples, and the 25 least frequent OTUs in ≤5 (10.4%) soil samples (Supplementary Fig. S2a). The first eight abundant OTUs (each >200 reads) accounted for 61.4% of the total AM fungal reads, and the remaining 65 OTUs accounted for 38.6% (Supplementary Fig. S2b). Among these 73 OTUs (Fig. 2), 33 belonged to Glomeraceae (14 *Glomus*, four *Funneliformis*, two *Sclerocystis*, two *Rhizophagus*, and 11 unidentified genus), nine to Gigasporaceae (nine *Scutellospora*), seven to Acaulosporaceae (seven *Acaulospora*), six to Ambisporaceae (six *Ambispora*), five to Claroideoglomeraceae (five *Claroideoglomus*), five to Diversisporaceae (two *Diversispora*, one *Redechera*, and two unidentified genus), four to Archaeosporaceae (four *Archaeospora*), two to Paraglomeraeae (two *Paraglomus*), one to Entrophosporaceae (one *Entrophospora*), and one to Pacisporaceae (one *Pacispora*). A rarefaction analysis showed that almost 16 rarefaction curves for observed AM fungal OTUs representing 16 treatments within four original elevations tended to reach the saturation platform, indicating that sequencing effort was sufficient to identify the most AM fungi in this study (Supplementary Fig. S3).

### AM fungal community

**Figure 3:**
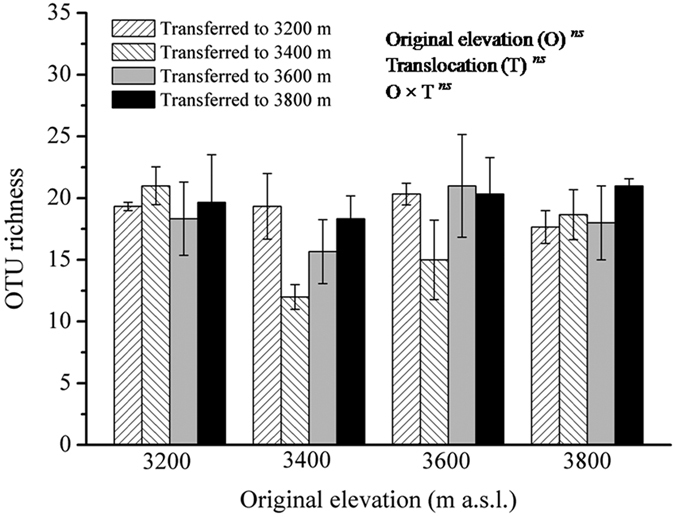
Arbuscular mycorrhizal (AM) fungal OTU richness under reciprocal translocation along an altitudinal gradient. Two-way ANOVA showed that original elevation (O), translocation (T) and their interactions (O × T) did not significantly affect AM fungal OTU richness (*ns*, *P *≥ 0.05). Data are mean ± SE (*n *= 3).

**Figure 4:**
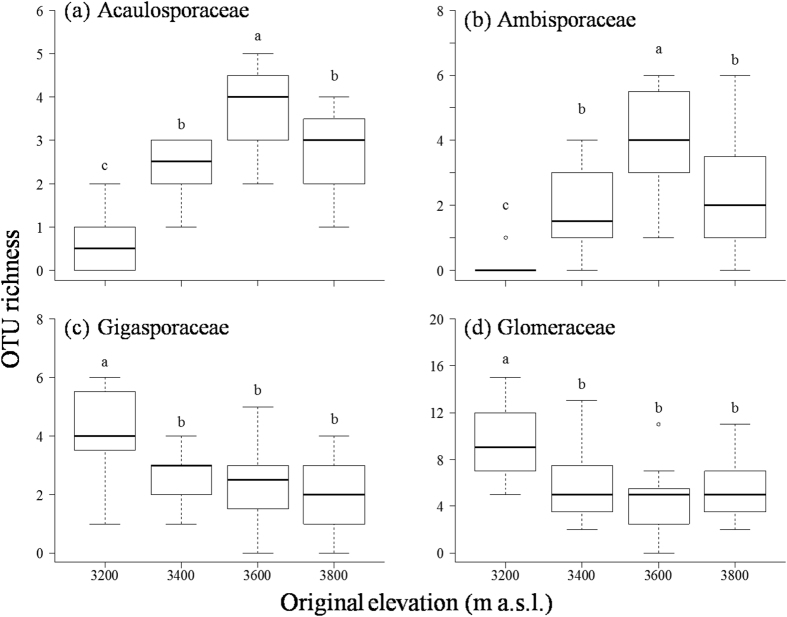
OTU richness of arbuscular mycorrhizal fungal families in different elevations. (**a**) Acaulosporaceae, (**b**) Ambisporaceae, (**c**) Gigasporaceae, and (**d**) Glomeraceae. The OTU richness of the each family was not significantly affected by translocation within each original elevation, data were thus pooled in each original elevation followed by Tukey’s HSD tests for Acaulosporaceae, Gigasporaceae and Glomeraceae (homogeneity of variance) or by pairwise comparisons for Ambisporaceae (heterogeneity of variance) to reveal the difference among four original elevations. Columns without shared letters indicate significant difference at *P* < 0.05.

AM fungal OTU richness ranged from 12 to 21 amongst all 16 treatments, and a two-way analysis of variance (ANOVA) revealed that original elevation, translocation and their interaction did not significantly affect AM fungal OTU richness (all *P *> 0.05; Fig. 3). However, original elevation, but not translocation had significant effect on the OTU richness of AM fungal families Acaulosporaceae (ANOVA: *F* = 24.980, *P *< 0.001), Ambisporaceae (Kruskal–Wallis test: *X*^2^ = 25.810, *P *< 0.001), Gigasporaceae (ANOVA: *F* = 6.997, *P *< 0.001), and Glomeraceae (ANOVA: *F* = 6.598, *P *< 0.001). For example, the OTU richness of Acaulosporaceae and Ambisporaceae were significantly lower at original 3,200 m than at original 3,400 m, 3,600 m and 3,800 m, but no significant difference between original 3,400 m and 3,800 m was observed (Fig. 4a,b). In contrast, the OTU richness of Gigasporaceae and Glomeraceae were significantly higher at original 3,200 m than at original 3,400 m, 3,600 m and 3,800 m, but no significant difference among 3,400 m, 3,600 m and 3,800 m was observed (Fig. 4c,d). In addition, indicator species analyses showed that in the 73 AM fungal OTUs, 12 (six Glomeraceae, five *Scutellospora*, and one *Redeckera*) were indicators in 3,200 m, two (*Glomus* and *Scutellospora*) in 3,400 m, six (four *Ambispora* and two *Acaulospora*) in 3,600 m, and four (*Acaulospora*, *Entrophospora*, *Pacispora*, and *Paraglomus*) in 3,800 m (*Indval* value >0.3, *P *< 0.05; Table 1).

**Figure 5:**
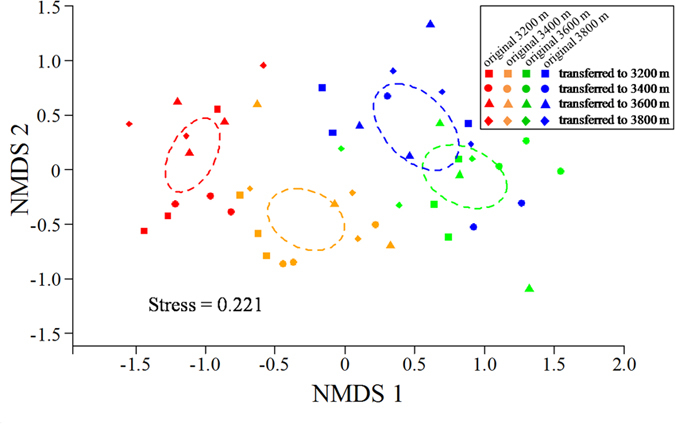
Non-metric multidimensional scaling (NMDS) of arbuscular mycorrhizal fungal community composition. Circles with dashed line in NMDS plot are 95% confidence ellipses of original elevations of 3200 m (red color), 3400 m (orange color), 3600 m (green color) and 3800 m (blue color).

**Figure 6:**
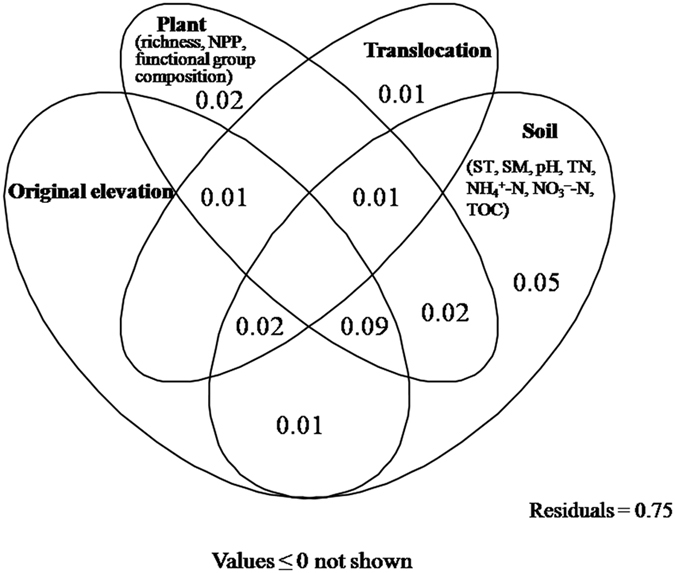
Variation partitioning analysis showing the effects of original elevation, translocation, soil and plant variables on arbuscular mycorrhizal fungal community. Numbers indicate the proportion of explained variation. Abbreviations: ST, soil temperature; SM, soil moisture; TN, soil total nitrogen; TOC; soil total organic carbon; richness, plant species richness; NPP, plant net primary production.

Permutational multivariate analysis of variance (PerMANOVA) showed that the AM fungal community composition was significantly affected by original elevation (*F* = 11.109, *R*^2^ = 0.193, *P *< 0.001), but not by translocation (*F* = 1.359, *R*^2^ = 0.024, *P* = 0.166) and interaction between original elevation and translocation (*F* = 1.240, *R*^2^ = 0.021, *P* = 0.229). Nonmetric multidimensional scaling (NMDS) analysis also showed that the AM fungal community composition was significantly affected by original elevation, but not by translocation (Fig. 5). Furthermore, partial Mantel tests showed that the AM fungal community composition was significantly related to plant functional group composition (*r* = 0.193, *P* = 0.011) and soil moisture (*r* = 0.172, *P* = 0.017) after translocation, and was marginally related to soil TOC (*r* = 0.123, *P* = 0.073), NH_4_^+^–N (*r* = 0.121, *P* = 0.075) and NO_3_^−^–N (*r* = 0.120, *P* = 0.083) (Table 2). Variation partitioning showed that total 25% of variation in AM fungal community composition was explained (Fig. 6). Of these, 20% of variation was explained by soil, 15% by plant, 13% by original elevation, and 5% by translocation (Fig. 6).

## Discussion

The AM fungal spore density was significantly higher at lower elevation than at higher elevation in this study. Similarly, a decrease trend in AM fungal spore density along an altitudinal gradient was found in another alpine meadow on the Qinghai-Tibet Plateau^6^ and in a mountainous grassland in Argentina^5^. Furthermore, we found that the AM fungal spore density was significantly correlated negatively with soil moisture and TOC but positively with plant species richness. In the study site, soil moisture and TOC were lower, but plant species richness was much higher at lower elevation compared with the higher elevation (Supplementary Table S1). These results suggested that AM fungal spore density may be affected by plant species diversity and soil moisture and TOC, as reported in previous studies^37^,^38^,^39^,^40^.

By contrast, we found that the AM fungal spore density was not significantly influenced by translocation from original 3,400 m, 3,600 m and 3,800 m sites, respectively. Likewise, it was found that experimental warming had no significant effect on AM fungal spore density in the same alpine meadow^24^ and in a semiarid steppe in north China^27^. However, the AM fungal spore density was significantly increased by translocation from original 3,200 m to 3,400 m and 3,600 m in this study. This finding may be because some AM fungi, such as *Acaulospora* and *Ambispora* had high abundance at 3400 m and 3600 m sites in this study (Table 1). Similarly, some previous studies reported that some AM fungi (*e.g. Acaulospora alpina* and *Gigaspora gigantea*) are known to be active under cooler but dormant during warmer conditions^41^,^42^, or some AM fungi are sensitive to temperature decline and thereby they would have to produce more spores for proliferation and survival in extreme conditions^43^,^44^. By contrast, AM fungal spore density was not significantly affected by translocation from low original sites (3,200 m, 3,400 m and 3,600 m) to high site (3,800 m) in this study. It may be interpreted that with increasing elevation some AM fungi (*e.g. Ambispora*, *Glomus*, *Redeckera*, and *Scutellospora*) in abundance were decreased, but others (*e.g. Entrophospora*, *Pacispora*, and *Paraglomus*) in abundance were increased (Table 1), which may result in no significant changes in AM fungal spore density in this study.

The AM fungal OTU richness was not significantly affected by original elevation, downward translocation and upward translocation in the current study. Consistently, there was no significant difference in AM fungal OTU richness within roots of *Kobresia* sp. in the different elevations on the Qinghai-Tibet Plateau^45^. It was also found that a three-year warming did not significantly affect AM fungal OTU richness in the same alpine ecosystem^24^. However, the OTU richness of families Acaulosporaceae and Ambisporaceae increased with increasing altitude. Furthermore, indicator species analyses showed that the abundance of seven OTUs (OTU3, OTU20, OTU21, OTU31, OTU35, OTU59, and OTU68) of these two families was higher at 3,600 m and 3,800 m than at 3,200 m and 3,400 m in this study. Similarly, compared with the lower elevation, *Acaulospora* fungi of Acaulosporaceae were relatively more abundant at higher elevation sites in Swiss Alps^42^ and in the Qinghai-Tibet Plateau^6^,^16^. In fact, *Ambispora fennica*, the representative species of Ambisporaceae, was initially isolated from a subarctic region in Finland (62°30′N)^46^, and Ambisporaceae fungi were previously found to be more abundant in higher elevation than in lower elevation regions on the Qinghai-Tibet Plateau^16^,^47^. These results suggested that members of Acaulosporaceae and Ambisporaceae tend to occur in relatively cold habitats. However, the richness of Acaulosporaceae and Ambisporaceae went down at the highest elevation of 3,800 m in this study, suggesting that extremely environmental stress may suppress the growth and survival of these fungi^6^.

By contrast, we observed that the OTU richness of families Gigasporaceae and Glomeraceae was significantly higher at lower elevation than at higher elevation sites. Meanwhile, indicator species analyses showed that the abundance of 12 OTUs of these two families was significantly higher at 3,200 m than at >3,400 m in this study. Similarly, Gigasporaceae fungi were mainly found at 3,300 m and 3,500 m, but unable to be detected above 3,700 m in the Puna mountain grassland in Argentina^5^ and mainly detected in <3,000 m but not in >4,000 m on the Qinghai-Tibet Plateau^6^. In addition, although Glomeraceae was dominant in this study as reported in previous studies^6^,^7^,^8^,^48^, the species richness of Glomeraceae also declined with increasing altitude^6^. These findings suggested that members of Gigasporaceae and Glomeraceae prefer to relatively lower elevation compared with the highland.

The AM fungal community composition was significantly affected by original elevation in the present study. The distinct AM fungal community compositions were also found along altitudinal gradients on the basis of analysis of spore morphology^6^ and molecular techniques^8^,^47^. Furthermore, we found that the AM fungal community composition was significantly related to soil moisture and plant functional group composition, and was marginally related to soil TOC and available N. Indeed, these plant and soil variables showed substantial difference amongst different original elevations in this study (Supplementary Table S1), which may result in changes in AM fungal composition, as previous studies reported that AM fungal community composition was structured by plant community^11^,^24^,^49^ and soil variables^13^,^14^,^15^,^27^,^50^. Nevertheless, we found that the AM fungal community composition was not significantly influenced by the downward or upward translocation. Similarly, the AM fungal community composition was not significantly affected by one-year warming in a grassland in UK^28^ and by five-year warming in a semiarid steppe in northern China^26^.

In conclusion, the AM fungal spore density was significantly affected by original altitude but not by two-year reciprocal translocation, except for a significant increase by translocation from original 3,200 m to 3,400 m and 3,600 m sites. The OTU richness of AM fungi was not significantly affected by original elevation and reciprocal translocation. However, with increasing altitude the OTU richness of Acaulosporaceae and Ambisporaceae increased, whereas this parameter of Gigasporaceae and Glomeraceae declined generally. The AM fungal community composition was significantly shaped by original elevation, but not by reciprocal translocation. Our results suggested that original elevation, rather than short-term reciprocal translocation is a strong determinant in structuring AM fungal community in the alpine meadow on the Qinghai-Tibet Plateau. However, we realize that only two-year experiment was carried out in this study, which may be not completely reflecting the effect of long-term global climate change on mycorrhizal community, thus a longer experimental period should be needed to better unveil this issue in further study.

## Methods

### Experimental design and sampling

The study was conducted at the Haibei Alpine Meadow Ecosystem Research Station (HAMERS) of the Chinese Academy of Sciences, along an altitudinal gradient (3,200 m, 3,400 m, 3,600 m, and 3,800 m above sea level) in the south slope of Qilian Mountains on the Qinghai-Tibet Plateau, China. The climate at HAMERS is highland continental, characterized to be cold and long in winter but warm and short in summer. This experiment was established in 2007 by Wang *et al.*^51^. Briefly, four 20 m long × 8 m wide plots were fenced at 3,200 m (37°36′42.3″N, 101°18′47.9″E), 3,400 m (37°39′55.1″N, 101°19′52.7″E), 3,600 m (37°41′46.0″N, 101°21′33.4″E), and 3,800 m (37°42′17.7″N, 101°22′09.2″E) sites. These four sites included different plant communities within 9 km of one another. The plant community at 3,200 m is dominated by *Kobresia humilis*, *Elymus nutans*, *Poa* spp., *Carex* spp., *Scripus distigmaticus*, *Gentiana straminea*, *G. farreri*, *Leontop odiumnanum*, and *Potentilla nivea*. The plant community at 3,400 m is dominated by alpine shrub *Potentilla fruticosa* and jointly by *Kobresia capillifolia*, *K. humilis*, and *Saussurea superba*. The plant community at 3,600 m site is dominated by *K. humilis*, *Saussurea katochaete*, *P. nivea*, *Thalictrum alpinum*, *Carex* spp., *Poa* spp., and *P. fruticosa*. The plant community at 3,800 m is dominated by *K. humilis*, *L. odiumnanum*, and *Poa* spp.^51^.

Twelve intact soil blocks (100 cm × 100 cm wide, 30–40 cm deep; *i.e.* 30 cm in depth at 3,800 m due to shallower soil layer) with attached vegetation were excavated from each of the four altitudes and reciprocally transferred across the altitudinal gradient after the soil started to thaw in early May 2007. Among the transferred soil blocks, three blocks from each altitude were also removed and then reinstated at the same site as control blocks that had been handled as similarly as possible as those blocks moved to other elevations. As such, there were three replicate transfers from each altitude, and these intact soil blocks were fully randomized throughout the study site. The distance between adjacent blocks was *ca*. 0.6 m in each plot.

On 2nd August 2009, three soil cores (30 cm in depth, 1.8 cm in diameter) were randomly collected from each block and mixed as one composite sample. A total of 48 soil samples were used in this study. The soil samples were immediately packed in an ice box and transported to our laboratory. Fresh soil samples were sieved through 1 mm sieve to remove roots and debris. Subsoil samples for DNA extraction were stored at −80 °C until analysis. Subsoil samples for AM fungal spore density were air-dried and stored at 4 °C until analysis. Subsoil samples for soil variables including pH, moisture, temperature, TOC, total N, NO_3_^−^–N and NH_4_^+^–N were determined by Yang *et al.*^52^. Plant variables including species richness, functional group composition, and net primary production (NPP) were determined by Wang *et al.*^32^. The information on the plant and soil variables is presented in Supplementary Table S1.

### AM fungal spore density

AM fungal spores were extracted from 20.0 g air-dried soil of each sample with deionized water using the wet-sieving and decanting method^53^ and then counted under a microscope (Nikon 80i, Tokyo, Japan) at 40 × magnification.

### DNA extraction, PCR and 454 pyrosequencing

Genomic DNA was extracted from 0.5 g frozen soil using a direct bead-beating extraction method with a PowerSoil DNA isolation kit (MoBio Laboratories, Inc. USA) according to the manufacturer’s instruction. Genomic DNA for 454 pyrosequencing was amplified using a two-step PCR procedure. The first amplification with primers GeoA-2^54^ and NS4^55^ was carried out in a final 25 μL reaction solution including 2.5 μL 10 × buffer, 1.5 mM MgCl_2_, 200 μM of each dNTP, 0.75 μM of each primer, 1.5 U *Taq* polymerase (Takara, Japan), and *ca.* 10 ng of template DNA combined with sterile deionized water. The thermal cycling was followed by an initial denaturation at 94 °C for 5 min, 35 cycles of denaturation at 94 °C for 45 s, annealing at 54 °C for 1 min, and extension at 72 °C for 1.5 min, followed by a final extension at 72 °C for 10 min. The product of the first amplification was diluted with sterilized deionized water by a factor of 20 and 1.0 μL diluted solution was used as the template for the nested PCR. Conditions for the nested PCR were similar to the first PCR, except for 58 °C annealing temperature, 30 cycles, and primers NS31^56^ and AML2^57^ linked to sequencing adaptors A and B, respectively (Supplementary Table S2). Barcode sequence, 10 bases in length, was inserted between the A adaptor and NS31 primer sequence. The nested PCR products were then loaded on a 1% agarose gel (Biowest, Spain) with 1.0 × TAE buffer (40 mM Tris base, 20 mM glacial acetic acid, 1 mM EDTA, pH 8.0), visualized after Goldview staining (Applied Biosystems, USA) under ultraviolet light, and then purified using an Axygen PCR product gel purification kit (Axygen, California, USA). The purified PCR products were measured using a fluorescence spectrophotometer (TBS 380, Promega, USA), and 50 ng of DNA from each of the 48 samples were pooled and adjusted to 10 ng μL^−1^. The pooled product was subjected to 454 pyrosequencing on a Roche Genome Sequencer FLX Titanium (454 Life Sciences, Branford, CT, USA). The representative 18S rDNA sequences obtained in this study have been submitted to the European Molecular Biology Laboratory (EMBL) nucleotide sequence database with the accession numbers LT576043–LT576115.

### Bioinformatics analysis

The noise generated during sequencing process was removed using the shhh.flow command in Mothur 1.31.2^58^. Subsequently, the denoised sequences with no valid primer sequence or DNA tag, containing ambiguous bases, homopolymers >8 bases, or with an average quality score <25 were removed using the “trim.seqs” command in Mothur. As the average read quality score dropped below 25 after 420^th^ base pair (bp), the remaining longer sequences were chopped to 400 bp to assure read quality^58^. Potential chimeras for the sampled sequences were checked with the ‘chimera.uchime’ command in Mothur. The non-chimeric sequences were clustered into different OTUs at a 97% similarity level based on the UPARSE pipeline using the USEARCH v8.0 after dereplication and singleton discarding^59^. The representative sequence of each OTU was identified by a basic local alignment search tool (BLAST) search against the National Center for Biotechnology Information (NCBI) nt database. The non-AM fungal OTUs identified on the basis of the closest BLAST hit (the first top 50 references with the highest sequence similarity) not annotated as ‘Glomeromycota’ were removed from the dataset. The representative sequences of AM fungal OTUs were confirmed by BLAST against the Maarj*AM* database^60^. The number of sequence per sample was normalized to the smallest sample size by using the ‘sub.sample’ command in Mothur to account for the influence of different read numbers on the analysis of AM fungal community. A neighbor-joining tree of the representative OTU sequences obtained in this study and the reference sequences downloaded from GenBank was constructed by using the *p*-distance model with 1,000 replicates to produce bootstrap values in MEGA 5 61 and thus identify the taxonomic position of the obtained AM fungal OTUs. In order to assess the efficiency of the sequencing, rarefaction curves of the observed AM fungal OTUs were generated for each treatment (including three replicates) using the *EstimateS* v.9^62^.

### Statistical analysis

AM fungal spore density is defined as spore numbers per gram air dried soil in a sample. The frequency of a specified AM fungal OTU is defined as the percentage of the number of samples where this OTU observed to the number of all samples. The abundance of a given AM fungal OTU is defined as the read numbers of that OTU in a sample. The relative abundance of a specific AM fungal OTU is defined as percentage of the number of reads where OTU was detected to the number of all reads in a sample. AM fungal OTU richness is defined as OTU numbers in a sample, and the richness of a given family is all OTU numbers of that family in a sample. A two-way ANOVA was used to examine the effects of original elevation, translocation and their interaction on AM fungal spore density and richness if the data satisfied the normality of distribution and homogeneity of variance amongst 16 treatments before and after sqrt and log transformation were carried out. Significant differences between treatments were further compared using Tukey’s honest significant difference (HSD) tests at *P *< 0.05. For the data that did not satisfy homogeneity of variance amongst treatments, nonparametric Kruskal–Wallis test was applied to examine the effect of original elevation and translocation. Moreover, if AM fungal variables did not significantly differ among the translocation treatments within each original elevation, these data were pooled in accordance with original elevation and then subjected to Tukey’s HSD tests (homogeneity of variance) or pairwise comparisons (heterogeneity of variance) at *P *< 0.05 to reveal the difference among original elevations. In order to determine AM fungal indicator species for each original elevation, we conducted indicator species analysis (species with *Indval* values > 0.3 and *P *< 0.05, are strong indicators)^63^ based on OTU relative abundance using the function ‘indval’ in the labdsv^64^ package in R^65^.

The distance matrices of AM fungal community composition (based on OTU relative abundance), soil (temperature, moisture, pH, TOC, total N, NO_3_^−^–N, and NH_4_^+^–N), and plant (species richness, functional group composition, and NPP) were constructed by calculating dissimilarities using the Bray–Curtis method^66^. PerMANOVA was carried out in the vegan^67^ package in R^65^ to evaluate the effects of original elevation, translocation and their interaction on AM fungal community composition. Subsequently, the AM fungal community composition was ordinated using NMDS with the dissimilarity matrices using the ‘metaMDS’ function in the vegan^67^ package in R^65^. Mantel tests were applied to explore correlations between pairs of dissimilarity matrices, partial Mantel tests to explore the independent effects of every soil and plant variables on the AM fungal community composition in the ecodist^68^. Moreover, the varpart function in the vegan^67^ package in R^65^ was used to partitioning the variation of AM fungal community dissimilarity by original elevation, translocation, soil (temperature, moisture, pH, TOC, total N, NO_3_^−^–N, and NH_4_^+^–N), and plant (species richness, NPP, and functional group composition) variables.

## Additional Information

**Accession codes:** European Molecular Biology Laboratory nucleotide sequence database with the accession numbers LT576043–LT576115.

**How to cite this article**: Yang, W. *et al.* Arbuscular mycorrhizal fungal community composition affected by original elevation rather than translocation along an altitudinal gradient on the Qinghai-Tibet Plateau. *Sci. Rep.* **6**, 36606; doi: 10.1038/srep36606 (2016).

**Publisher’s note**: Springer Nature remains neutral with regard to jurisdictional claims in published maps and institutional affiliations.

## Supplementary Material

Supplementary Information

## Figures and Tables

**Table 1:** Arbuscular mycorrhizal fungal indicator OTUs in different original elevations along an altitudinal gradient on the Qinghai-Tibet Plateau.

No.	OTU	Family	Genus	Original elevation	Indval value (>0.3)	*P* value
1	OTU73	Gigasporaceae	*Scutellospora*	3200	0.872	0.001
2	OTU14	Gigasporaceae	*Scutellospora*	3200	0.611	0.001
3	OTU27	Gigasporaceae	*Scutellospora*	3200	0.402	0.005
4	OTU32	Glomeraceae	*Glomus*	3200	0.397	0.004
5	OTU12	Diversisporaceae	*Redeckera*	3200	0.395	0.004
6	OTU38	Glomeraceae	unidentified	3200	0.389	0.004
7	OTU48	Glomeraceae	unidentified	3200	0.381	0.003
8	OTU71	Glomeraceae	*Glomus*	3200	0.340	0.028
9	OTU5	Gigasporaceae	*Scutellospora*	3200	0.333	0.010
10	OTU30	Gigasporaceae	*Scutellospora*	3200	0.333	0.017
11	OTU42	Glomeraceae	unidentified	3200	0.316	0.035
12	OTU62	Glomeraceae	unidentified	3200	0.308	0.015
13	OTU53	Acaulosporaceae	*Acaulospora*	3400	0.821	0.001
14	OTU26	Glomeraceae	*Glomus*	3400	0.461	0.001
15	OTU3	Acaulosporaceae	*Acaulospora*	3600	0.627	0.001
16	OTU31	Ambisporaceae	*Ambispora*	3600	0.569	0.001
17	OTU68	Ambisporaceae	*Ambispora*	3600	0.459	0.001
18	OTU21	Acaulosporaceae	*Acaulospora*	3600	0.443	0.001
19	OTU59	Ambisporaceae	*Ambispora*	3600	0.402	0.013
20	OTU35	Ambisporaceae	*Ambispora*	3600	0.316	0.017
21	OTU10	Pacisporaceae	*Pacispora*	3800	0.543	0.001
22	OTU20	Acaulosporaceae	*Acaulospora*	3800	0.508	0.001
23	OTU28	Paraglomeraceae	*Paraglomus*	3800	0.361	0.010
24	OTU72	Entrophosporaceae	*Entrophospora*	3800	0.360	0.010

**Table 2:** Mantel and partial Mantel tests of the arbuscular mycorrhizal fungal community with soil and plant variables.

Variable	Mantel test		Partial mantel test	
	*r* value	*P* value	*r* value	*P* value
Soil temperature	−0.022	0.726	−0.095	0.997
Soil moisture	0.147	0.033	0.172	0.017
Soil pH	0.038	0.260	0.075	0.157
Soil total organic carbon	0.190	0.012	0.123	0.073
Soil total nitrogen (N)	0.171	0.030	0.066	0.172
Soil NO_3_^−^–N	0.119	0.086	0.120	0.083
Soil NH_4_^+^–N	0.156	0.042	0.121	0.075
Plant species richness	−0.029	0.633	−0.075	0.864
Plant net primary production	−0.013	0.505	−0.086	0.835
Plant functional group composition	0.166	0.015	0.193	0.011
