# Supplementary material for: Arbuscular mycorrhizal fungal community composition affected by original elevation rather than translocation along an altitudinal gradient on the Qinghai-Tibet Plateau
Source: Sci Rep. 2016 Nov 9;6:36606. doi: 10.1038/srep36606 (PMC5101527; doi:10.1038/srep36606)
Supplement: Supplementary Information [file srep36606-s1.pdf]

Supplementary Information

Title: Arbuscular mycorrhizal fungal community composition affected by original elevation rather than translocation along an altitudinal gradient on the Qinghai-Tibet Plateau

Author list: Wei Yang[1,2#], Yong Zheng[1#], Cheng Gao[1], Ji-Chuang Duan[3], Shi-Ping Wang[4] & Liang-Dong Guo[1,5]*

[1]State Key Laboratory of Mycology, Institute of Microbiology, Chinese Academy of Sciences, Beijing 100101, China. [2]College of Resources and Environment, Northeast Agricultural University, Harbin 150030, China. [3]Binhai Research Institute in Tianjin, Tianjin 300457, China. [4]Laboratory of Alpine Ecology and Biodiversity, Institute of Tibetan Plateau Research, Chinese Academy of Sciences, Beijing 100101, China. [5]College of Life Sciences, University of Chinese Academy of Sciences, Beijing 100049, China.

[#] Wei Yang and Yong Zheng contributed equally

*Corresponding author: Prof. Dr. Liang-Dong Guo

State Key Laboratory of Mycology, Institute of Microbiology, Chinese Academy of Sciences, Beijing 100101, China.

Tel.: +86 10 64807510

E-mail: guold@im.ac.cn (L.D. Guo)

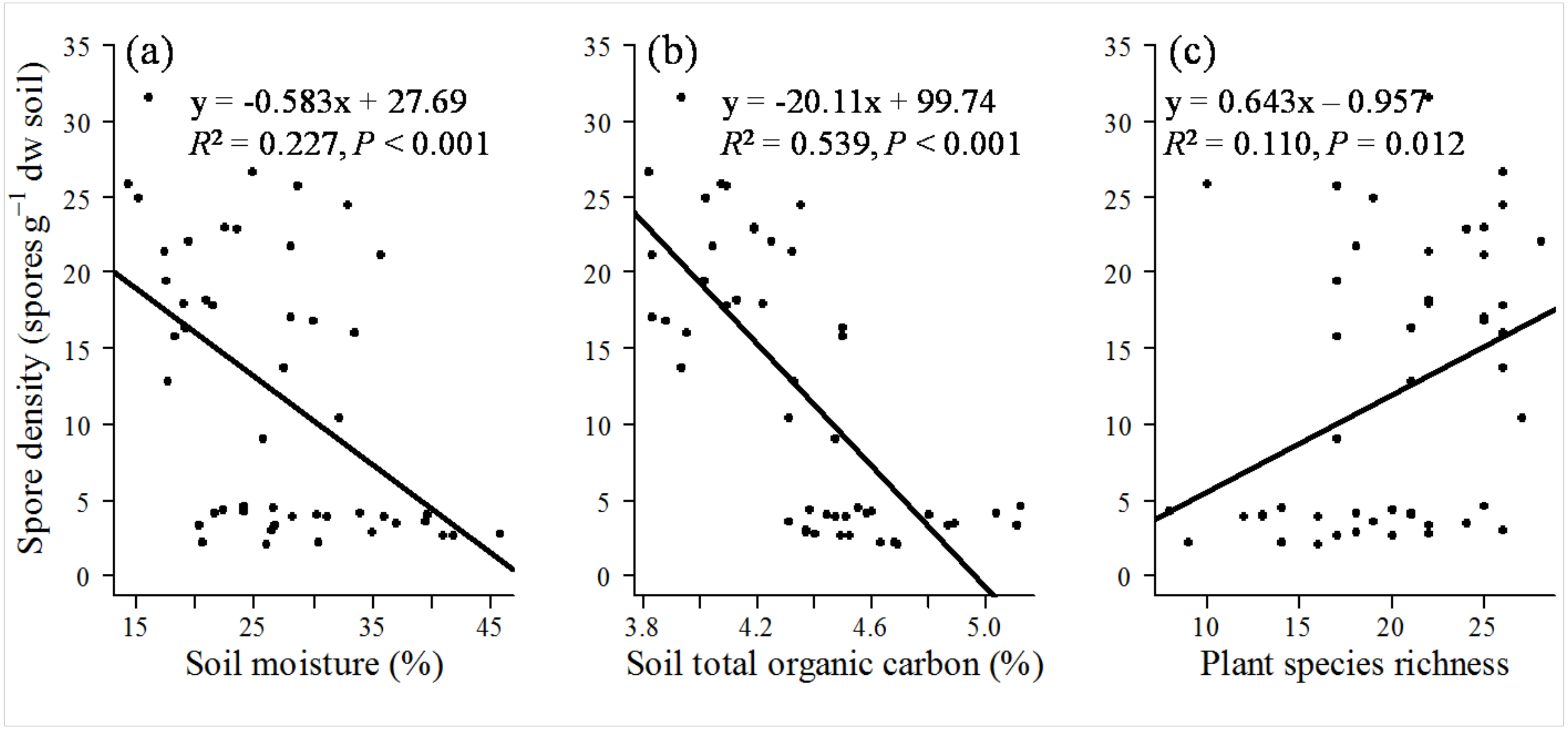

Figure S1. Linear regressions of arbuscular mycorrhizal fungal spore density versus soil and plant variables. (**a**) soil moisture, (**b**) soil total organic carbon and (**c**) plant species richness.

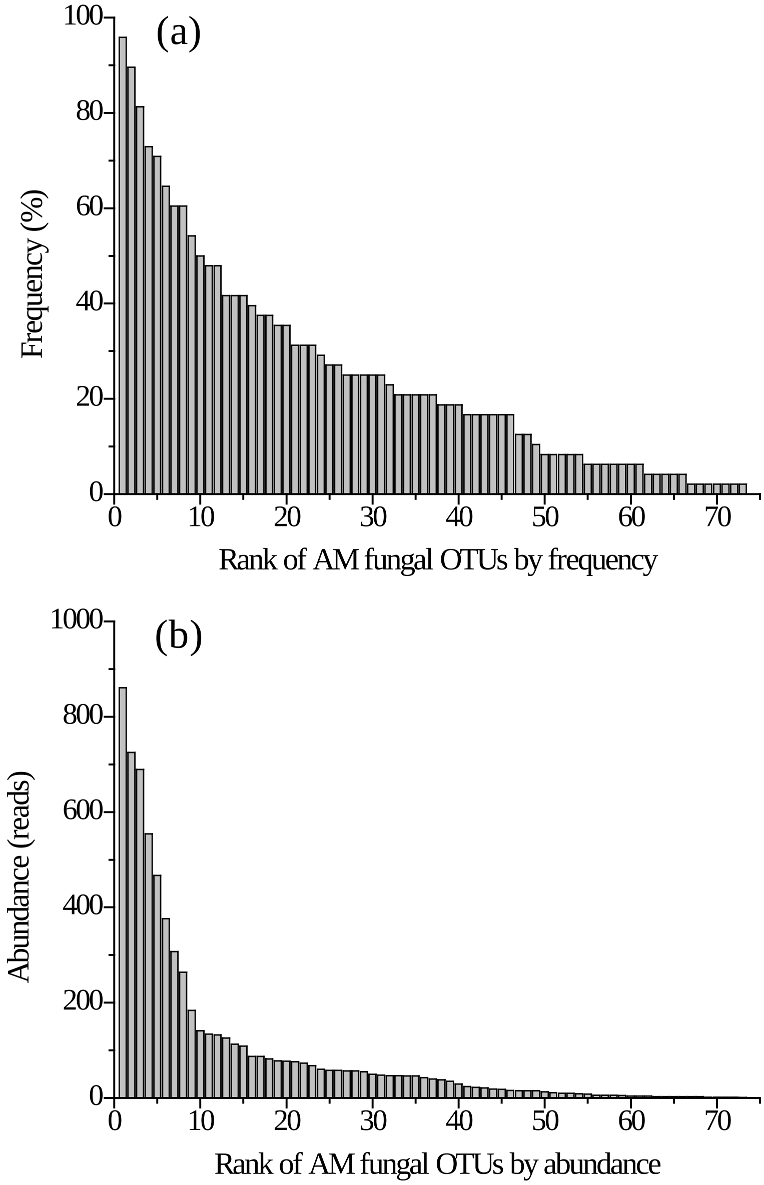

Figure S2. Arbuscular mycorrhizal (AM) fungal OTUs. (**a**) Rank by frequency and (**b**) by abundance in an alpine meadow on the Qinghai-Tibet Plateau.

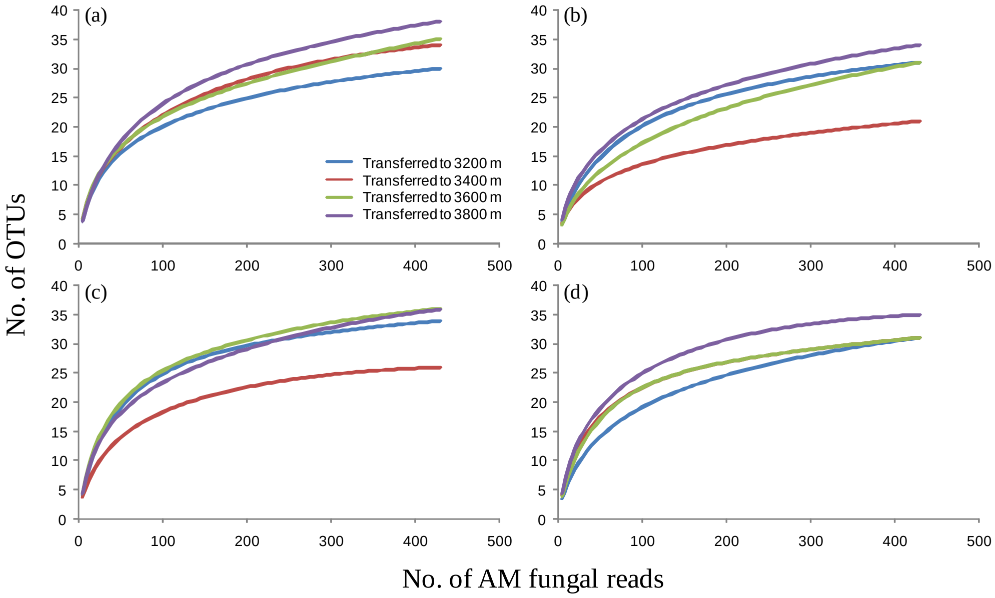

Figure S3. Rarefaction curves for observed arbuscular mycorrhizal fungal OTUs. (**a**) Original elevation of 3200 m, (**b**) Original elevation of 3400 m, (**c**) Original elevation of 3600 m and (**d**) Original elevation of 3800 m.

Table S1. Soil and plant variables amongst treatments in this study

Treatment (origin-destination)	ST (^oC)	SM (%)	pH	TOC (g kg^{-1})	TN (g kg^{-1})	NO_3^-–N (mg kg^{-1})	NH_4^+–N (mg kg^{-1})	Richness	NPP (g m^{-2})	Cover of plant functional groups (%)			
										Legumes	Grasses	Sedges	Forbs
3200-3200	12.43±0	22.4±8.4	7.9±0.1	4.3±0	3.7±0	0.9±0.3	3.7±0.7	23.3±3.2	535.4±78.3	21.7±9.6	55±6.2	9.67	72±29.5
3200-3400	9.15±0	20.3±2	7.8±0.2	4.2±0	3.6±0.1	1±0.3	3.4±1.6	25±3	450.3±72.3	17.7±5.7	77.7±10	11.00	50.3±7.1
3200-3600	8.88±0	23.7±8.1	7.5±0.1	4.1±0	3.7±0.1	1.5±0.2	3.5±1.3	15±4.4	175.7±20.3	2±1.7	19.3±8.1	15.33	48.7±3.2
3200-3800	6.84±0	31.2±3.9	7.5±0.2	3.8±0	3.3±0	1±0.1	4.8±1.6	25±0	323±37.7	16±7	67.3±18.6	12.67	47±23.1
3400-3200	12.43±0	23.4±8.2	8±0	4.5±0.1	3.9±0	0.9±0.2	1.3±0.2	21.3±4.5	385.9±95	6.3±5.8	45.3±6.4	6.00	80.3±6.8
3400-3400	9.15±0	21.9±1.4	7.7±0.1	4.1±0.1	3.8±0	0.8±0.1	2.1±0.9	24±2	326.2±31.2	1.3±2.3	51.7±25.7	8.33	76±20.8
3400-3600	8.88±0	16.3±1.1	7.6±0.2	4±0	3.9±0	1.1±0.3	2.4±0.5	19.3±2.5	131.7±18.7	1.3±1.5	18.7±7.6	13.33	37.7±5
3400-3800	6.84±0	28.5±4.4	7.7±0.1	3.9±0.1	3.7±0	0.8±0.2	1±0.2	26±0	185.8±38.4	4.3±1.5	33.3±15.9	13.00	58.7±15.4
3600-3200	12.43±0	26.1±7	7.8±0.1	5.1±0	4.4±0	1.1±0.3	3.5±1.5	22.7±2.1	281.9±31	7±1	44.7±4	34.33	83.3±8
3600-3400	9.15±0	34.4±6.8	7.6±0.1	4.9±0	4.2±0	0.9±0.4	2.7±1.3	22.3±1.5	237.1±32.5	2±1	8.7±3.8	65.67	64±12.8
3600-3600	8.88±0	25.6±4.9	7±0.1	4.7±0	4.1±0	1.6±0.3	2±0.3	13±3.6	139.9±20.8	3±2	1±1.7	40.33	22±19.7
3600-3800	6.84±0	42.7±2.5	7.2±0.2	4.5±0.1	4±0	1.4±0.2	2.4±0.9	19.7±2.5	160.7±52.7	2.3±1.2	13±3.6	32.33	58.3±22.3
3800-3200	12.43±0	24.1±2.4	7.7±0.2	4.6±0	4.3±0	1.2±0	1.4±0.7	13.3±5	274.2±29.9	3.7±1.2	22.7±15.9	42.33	57±25.4
3800-3400	9.15±0	26.1±3.9	7.2±0.1	4.4±0	4±0	0.8±0.5	1.3±0.7	16.7±3.5	248.6±20.3	3.7±3.5	10±3.5	59.67	64.7±10.1
3800-3600	8.88±0	33.6±6.5	7.5±0.2	4.4±0	4.2±0	1.8±0.6	1.3±0.7	21±4.4	176.9±33.3	10.3±11.4	13±14.8	44.67	17.3±9.5
3800-3800	6.84±0	31.7±3.9	7.2±0.1	4.5±0	4±0	1.4±0.2	1.5±0.4	13.7±2.1	211.1±65.2	1±1	2.3±2.3	69.67	15.3±6.5

Abbreviations: 3200-3200, 3200-3400, 3200-3600 and 3200-3800 mean transferred from original 3200 m to 3200 m, 3400 m, 3600 m and 3800 m. 3400-3200, 3400-3400, 3400-3600 and 3400-3800 mean transferred from original 3400 m to 3200 m, 3400 m, 3600 m and 3800 m. 3600-3200, 3600-3400, 3600-3600 and 3600-3800 mean transferred from original 3600 m to 3200 m, 3400 m, 3600 m and 3800 m. 3800-3200, 3800-3400, 3800-3600 and 3800-3800 mean transferred from original 3800 m to 3200 m, 3400 m, 3600 m and 3800 m. ST, soil temperature; SM, soil moisture; TN, soil total nitrogen; TOC; soil total organic carbon; NPP, plant (aboveground) net primary production; richness, plant species richness.

Table S2. Barcode sequence of each treatment in the raw sequence data used in this study

Sample No.	Treatment (origin-destination)	Primer name	Barcode sequence
Y-1	3200-3200	454_RL14_NS31	AGTACGAGAG
Y-2	3200-3200	454_RL19_NS31	ATAGTATACG
Y-3	3200-3200	454_RL24_NS31	CGTACGTCGA
Y-4	3200-3400	454_RL25_NS31	CTACTCGTAG
Y-5	3200-3400	454_RL30_NS31	ACACTCATAC
Y-6	3200-3400	454_RL36_NS31	ACGAGCGCGC
Y-7	3200-3600	454_RL37_NS31	ACGATGAGTG
Y-8	3200-3600	454_RL42_NS31	ACTAGTGATA
Y-9	3200-3600	454_RL47_NS31	ACTGCTGTAC
Y-10	3200-3800	454_RL49_NS31	AGACACTCAC
Y-11	3200-3800	454_RL54_NS31	AGATACGCTG
Y-12	3200-3800	454_RL59_NS31	AGCTAGATAC
Y-13	3400-3200	454_RL16_NS31	AGTAGACGTC
Y-14	3400-3200	454_RL18_NS31	AGTGTAGTAG
Y-15	3400-3200	454_RL21_NS31	CGACGACGCG
Y-16	3400-3400	454_RL28_NS31	GTGTACGACG
Y-17	3400-3400	454_RL31_NS31	ACAGACAGCG
Y-18	3400-3400	454_RL33_NS31	ACAGAGACTC
Y-19	3400-3600	454_RL40_NS31	ACGTCGCTGA
Y-20	3400-3600	454_RL41_NS31	ACGTCTAGCA
Y-21	3400-3600	454_RL46_NS31	ACTGATCTCG
Y-22	3400-3800	454_RL52_NS31	AGAGTACAGA
Y-23	3400-3800	454_RL55_NS31	AGATCTAGTC
Y-24	3400-3800	454_RL58_NS31	AGCGTGTGCG
Y-25	3600-3200	454_RL13_NS31	AGACTCGACG
Y-26	3600-3200	454_RL20_NS31	CAGTACGTAC
Y-27	3600-3200	454_RL22_NS31	CGACGAGTAC
Y-28	3600-3400	454_RL26_NS31	GTACAGTACG
Y-29	3600-3400	454_RL32_NS31	ACAGACTATA
Y-30	3600-3400	454_RL35_NS31	ACAGTGTCGA
Y-31	3600-3600	454_RL39_NS31	ACGCTCTCTC
Y-32	3600-3600	454_RL44_NS31	ACTCACTAGC
Y-33	3600-3600	454_RL45_NS31	ACTCTATATA
Y-34	3600-3800	454_RL50_NS31	AGACATATAG
Y-35	3600-3800	454_RL56_NS31	AGCAGCGTAG
Y-36	3600-3800	454_RL57_NS31	AGCGCACGAG

Y-37	3800-3200	454_RL15_NS31	AGTACTACTA
Y-38	3800-3200	454_RL17_NS31	AGTCGTACAC
Y-39	3800-3200	454_RL23_NS31	CGATACTACG
Y-40	3800-3400	454_RL27_NS31	GTCGTACGTA
Y-41	3800-3400	454_RL29_NS31	ACACAGTGAG
Y-42	3800-3400	454_RL34_NS31	ACAGCTCGTG
Y-43	3800-3600	454_RL38_NS31	ACGCGAGAGA
Y-44	3800-3600	454_RL43_NS31	ACTCACACTG
Y-45	3800-3600	454_RL48_NS31	ACTGTAGCGC
Y-46	3800-3800	454_RL51_NS31	AGACGTGATC
Y-47	3800-3800	454_RL53_NS31	AGAGTATCTC
Y-48	3800-3800	454_RL60_NS31	AGCTGTCGAC

Abbreviations: 3200-3200, 3200-3400, 3200-3600 and 3200-3800 mean transferred from original 3200 m to 3200 m, 3400 m, 3600 m and 3800 m. 3400-3200, 3400-3400, 3400-3600 and 3400-3800 mean transferred from original 3400 m to 3200 m, 3400 m, 3600 m and 3800 m. 3600-3200, 3600-3400, 3600-3600 and 3600-3800 mean transferred from original 3600 m to 3200 m, 3400 m, 3600 m and 3800 m. 3800-3200, 3800-3400, 3800-3600 and 3800-3800 mean transferred from original 3800 m to 3200 m, 3400 m, 3600 m and 3800 m.
